# Complete Genome Sequence of *Bacillus horikoshii* Strain 20a from Cuatro Cienegas, Coahuila, Mexico

**DOI:** 10.1128/genomeA.00592-17

**Published:** 2017-07-27

**Authors:** Eugenia Zarza, Luis D. Alcaraz, Bernardo Aguilar-Salinas, Africa Islas, Gabriela Olmedo-Álvarez

**Affiliations:** a Laboratorio de Biología Molecular y Ecología Microbiana, Departamento de Ingeniería Genética, Centro de Investigación y de Estudios Avanzados del Instituto Politécnico Nacional, Unidad Irapuato, Irapuato, Mexico; b Laboratorio Nacional de Ciencias de la Sostenibilidad, Instituto de Ecología, Universidad Nacional Autónoma de México, Mexico City, Mexico

## Abstract

We sequenced the *Bacillus horikoshii* 20a genome, isolated from sediment collected in Cuatro Cienegas, Mexico. We identified genes involved in establishing antagonistic interactions in microbial communities (antibiotic resistance and bacteriocins) and genes related to the metabolism of cyanophycin, a reserve compound and spore matrix material potentially relevant for survival in an oligotrophic environment.

## GENOME ANNOUNCEMENT

*Bacillus horikoshii* is an alkaliphilic, aerobic, endospore-forming bacterium, initially isolated from soil samples (1). We report the genome of *B. horikoshii* strain 20a isolated from shallow-water sediment collected in the Churince system in the Cuatro Cienegas Basin, Coahuila, Mexico (2). *Bacillus horikoshii* 20a has been studied to better understand how microbial assembly in communities is influenced by antagonistic interactions (2). Thus, it is important to identify genes involved in coping with this oligotrophic environment and genes related to defense responses. The 20a genome was sequenced with the PacBio RSII system, assembled with Canu v.1.3 (3), and circularized with Circlator v.1.3 (4). This assembly resulted in two contigs representing the chromosome (4,277,585 bp, 192.75× mean coverage) and an extrachromosomal feature (18,297 bp, 122.28× mean coverage). The contigs were annotated with RAST (5), which identified 24 rRNAs, 72 tRNAs, and 4,366 protein-coding sequences in 457 functional subsystems. Alignment of the 16S rRNA gene against the type strains in the RDP database (6) returned a 0.991 similarity score between *B. horikoshii* 20a and *B. horikoshii* DSM 8719. However, in the *B. horikoshii* 20a genome, there are 533 genes that are not found in any of the two *B. horikoshii* published genomes (strains DSM 8719 and FJAT-14233). RAST annotated 12 genes coding for bacteriocins and ribosomally synthesized antibacterial peptides, 48 genes related to resistance to antibiotics and toxic compounds, and 139 genes involved in dormancy and sporulation.

Interestingly, three genes located in tandem were annotated as genes for cyanophycin synthetase (CphA), and two other regions located 2.76 Mb upstream were identified as genes for cyanophycinase and isoaspartyl dipeptidase, respectively. These are enzymes involved in the metabolism of cyanophycin, a branched, nonribosomally synthesized polypeptide consisting of aspartic acid in the backbone and arginine in the side chain (7). Cyanophycin was originally described in *Cyanobacteria*, where it accumulates under conditions of intense light, high carbon dioxide concentration, or phosphate or sulfur starvation (8). Given its high nitrogen content and water insolubility, cyanophycin has been suggested to constitute a reserve material (9, 10). Cyanophycin synthetase has also been found in *Firmicutes*, like *Clostridium perfringens*, where it possibly plays a role in spore assembly as a matrix material giving spores their normal morphology (11). Both cyanophycin functions, as a nitrogen and carbon reserve and as a spore material, might be relevant for the survival of *B. horikoshii* 20a in its oligotrophic environment. We carried out PSI-BLAST (12) of one of the *B. horikoshii* 20a putative CphAs and evaluated the conservation of amino acid residues of the active sites for cyanophycin synthesis (13). An alignment including the three *B. horikoshii* 20a putative CphAs plus the PSI-BLAST results showed that the CphAs have the essential residues involved in the incorporation of aspartate but lack the essential residues for the incorporation of arginine. This composition is similar to those of cyanobacterial enzymes classified as CphA2 and CphA2′ (14). Cyanobacterial CphA2 contributes to the synthesis of cyanophycin in N_2_-fixing *Cyanobacteria*, where cyanophycin seems to play a role in nitrogen storage (15). Our transcriptome data show expression of the CphA, cyanophycinase, and isoaspartyl dipeptidase genes in *B. horikoshi* 20a.

### Accession number(s).

This genome has been deposited in GenBank under the GenBank accession numbers CP020880 and CP020881. The versions described in this paper are the first versions.
